# Platelets and Regulatory T Cells May Induce a Type 2 Immunity That Is Conducive to the Progression and Fibrogenesis of Endometriosis

**DOI:** 10.3389/fimmu.2020.610963

**Published:** 2020-12-14

**Authors:** Fengyi Xiao, Xishi Liu, Sun-Wei Guo

**Affiliations:** ^1^Shanghai OB/GYN Hospital, Fudan University, Shanghai, China; ^2^Shanghai Key Laboratory of Female Reproductive Endocrine-Related Diseases, Fudan University, Shanghai, China

**Keywords:** endometriosis, platelet, regulatory T cell, type 2 immunity, macrophage, fibrosis

## Abstract

Endometriosis is a hormonal disease, as well as a chronic inflammatory disease. While various immune cells are documented to be involved in endometriosis, there is a wanton lack of a bigger picture on how these cells are coordinated to work concertedly. Since endometriotic lesions experience cyclical bleeding, they are fundamentally wounds that undergo repeated tissue injury and repair (ReTIAR). In this study, we attempted to characterize the role of platelets and regulatory T cells (Tregs) in modulating the lesional immune microenvironment and its subsequent effects on lesional progression and fibrogenesis. Through two mouse experiments, we show that, by disrupting predominantly a type 2 immune response in lesional microenvironment, both platelets and Tregs depletion decelerated lesional progression and fibrogenesis, likely through the suppression of the TGF-β1/Smad3 and PDGFR-β/PI3K/Akt signaling pathways. In particular, platelet depletion resulted in significantly reduced lesional expression of thymic stromal lymphopoietin (TSLP), leading to reduced aggregation of macrophages and alternatively activated (M2) macrophages, and of Tregs, T helper 2 (Th2) and Th17 cells but increased aggregation of Th1 cells, in lesions, which, in turn, yields retarded fibrogenesis. Similarly, Tregs depletion resulted in suppression of platelet aggregation, and reduced aggregation of M2 macrophages, Th2 and Th17 cells but increased aggregation of Th1 cells, in lesions. Thus, both platelet and Tregs depletion decelerated lesional progression and fibrogenesis by disrupting predominantly a type 2 immunity in lesional microenvironment. Taken together, this suggests that both platelets and Tregs may induce a type 2 immunity in lesional microenvironment that is conducive to lesional progression and fibrogenesis.

## Introduction

Featuring the presence of endometrial tissue outside of the uterine cavity and often characterized by pelvic pain and subfertility or infertility, endometriosis is a common gynecological disease affecting women of reproductive age (1). Evidence accrued in the last few years indicates that, like eutopic endometrium, endometriotic lesions undergo cyclical bleeding and the ensuing tissue repair, and thus akin to wounds undergoing repeated tissue injury and repair (ReTIAR), leading ultimately to fibrosis through epithelial-mesenchymal transition (EMT), fibroblast-to-myofibroblast transdifferentiation (FMT), and smooth muscle metaplasia (SMM) (2–4). Along with the ReTIAR line, we have previously shown that platelets are critically involved in the development of endometriosis (3, 5–7). We also have shown that M2a macrophages are involved in the fibrogenesis of endometriosis (8). Through the looking glass of this ReTIAR, we can readily understand why deep endometriosis is more fibrotic than ovarian endometrioma and why lesions are resistant to medical treatment (9), and why many drugs that shined in preclinical studies but failed in clinical trials (10). The ReTIAR notion also helps to shed new light on the pathophysiology of endometriosis (11), and help devise novel diagnostics of endometriosis (12).

Tissue repair following injury is of vital importance to the survival of all living organisms. Tissue repair can be roughly divided into several distinct but somehow overlapping phases: hemostasis, inflammation, proliferation, and remodeling (13). In all phases, immune cells are actively involved. Platelets and macrophages belong to the innate immune system, but the role of adaptive immunity in the progression of endometriosis is not well understood. To counter the deleterious effect of inflammation, specialized subsets of lymphocytes are recruited into the wounding sites to avoid tissue damage inflicted by excessive inflammation (14). Of particular interest is the forkhead box protein 3 (FOXP3) positive regulatory T cells (Tregs), which are known to play an indispensable role in establishing and maintaining immunosuppression as well as tissue homeostasis (15). More importantly, it has been established that Tregs are involved in eliciting type 2 immunity that is implicated in various fibrotic disorders (16) and are very likely to be involved in fibrogenesis of endometriosis as well.

Type 2 immunity is characterized by the production of interleukin (IL)-4, IL-5, IL-9, IL-13, IL-25, and IL-33, as opposed to type 1 immunity, which features interferon γ (IFNγ), IL-2, tumor necrosis factor α (TNF-α), and IL-12 cytokines (16). Key cell types associated with type 2 immune responses, including T helper 2 (Th2) cells, eosinophils, mast cells, basophils, type 2 innate lymphoid cells (ILC2s), and IL-4- and IL-13-activated macrophages (M2), regulate tissue repair following injury. Nevertheless, when the type 2 cytokine-mediated repair process goes awry, they can also contribute to the development of pathological fibrosis in many different organs/tissues (16).

In endometriosis, there seem to be conflicting reports on the Th1/Th2 response (17–19), but a preponderance of data indicate that a Th2 immune response appears to be more predominant (18, 20, 21). Indeed, Th2 cytokines, or more generally, a type 2 immunity, is typically regarded as anti-inflammatory and reparative in tissue repair, and, when dysregulated, drives fibrogenesis (16). IL-25, IL-33, and thymic stromal lymphopoietin (TSLP) have been identified to be key initiators of this type 2 immunity (22). In particular, TSLP is viewed as a master switch in inducing type 2 immune response (23, 24).

TSLP belongs to the IL-2 cytokine family and is structurally related to IL-7 (25). TSLP promotes the differentiation of naïve T-cells into a Th2 phenotype and the secretion of various profibrotic factors, including IL-13 (26). Following injury, epithelial cell-derived TSLP activates local dendritic cells (DCs) to secrete CC chemokine ligand 22 (CCL22) and CCL17, promoting Th2 cell differentiation and propagating the type 2 immune response (16). Activated platelets can induce TSLP production by microvascular endothelial cells in an IL-1β dependent manner (27). Macrophages also can secrete TSLP to promote pulmonary fibrosis (28). In endometriosis, TSLP is expressed in both stromal and epithelial cells, and its concentration in serum and peritoneal fluid from women with endometriosis has been reported to be elevated (29). Both IL-1β and estrogen can stimulate the TSLP production in endometriotic stromal cells (29, 30).

Human platelets express functional TSLP receptors, which can be activated by TSLP to promote platelet activation (31). TSLP influences Tregs differentiation and development (32), and stimulates the proliferation of FOXP3+ Tregs in the peripheral tissues and thymus (33).

Viewed from the ReTIAR vista, it is only natural to speculate that a type 2 immune response should prevail in lesional microenvironment as endometriosis progresses. Precisely due to the progressive nature, it is imperative to investigate this possibility in a serially designed experiment, which can be feasible only in an animal study. We started from the perturbation by depleting platelets, which are shown to play a critical role in lesional development (3, 5, 34). Due to the important role that Tregs play in type 2 immunity, we also perturbed the lesional progression by depleting Tregs with and without platelet depletion. We hypothesized that platelet and/or Tregs depletion would disrupt the type 2 immune response in lesional progression, stalling lesional fibrogenesis. This study was undertaken to test this hypothesis.

## Materials and Methods

### Animals

A total of 120 7-week-old virgin female Balb/C mice were purchased from the SLAC Experimental Animal Company (Shanghai, China) and used for this study. All mice were maintained under controlled conditions with a cycle of 12:12 h light/dark and had access to food and water *ad libitum*.

All experiments were performed under the guidelines of the National Research Council’s Guide for the Care and Use of Laboratory Animals (35) and were approved by the Institutional Experimental Animals Review Board of Shanghai OB/GYN Hospital, Fudan University.

### Induction of Endometriosis

We used an established mouse model of endometriosis by induction through intraperitoneal injection of uterine fragments (36), which has been used in our previous studies (5). In brief, after 1 week of acclimatization, 7-week-old donor mice were initially injected with 100 μg/kg estradiol benzoate (Animal Medicine Factory, Hangzhou, China) intramuscularly, twice a week. One week later they were sacrificed and their uteri were harvested. The uterine tissues were seeded in a Petri dish containing warm sterile saline and divided longitudinally with scissors. Two uterine horns from each mouse were minced with scissors to make sure that the maximal diameter of the fragments was consistently smaller than 1 mm. Then the fragments were injected intraperitoneally to recipient mice. To eliminate potential bias, endometrial fragments from one or two donor mice were mixed and injected intraperitoneally to two or four mice, each from one of the two or four groups. The animals were sacrificed 5 weeks after induction.

### Mouse Experiment Protocol

**Figure 1:**
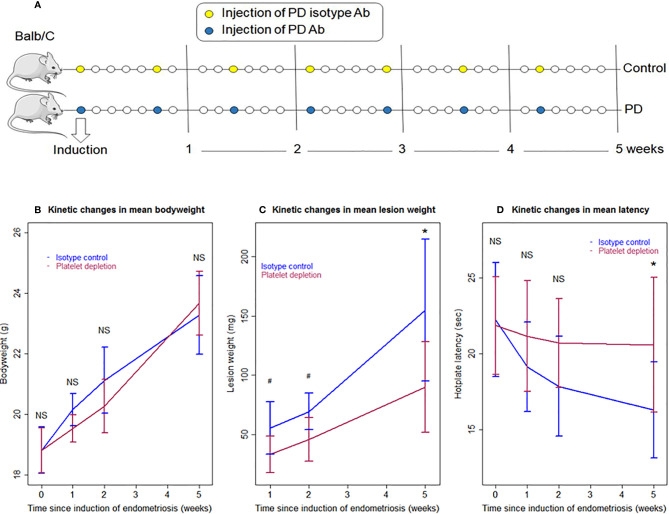
Schematic illustration of the experimental design, kinetics of bodyweight, lesion weight, and hotplate latency of the two groups of mice in Experiment 1. **(A)** Schematic illustration of the experimental design. The yellow circles represent the injection of the mock isotype antibody for the anti-platelet antibody, and the blue circles represent the injection of anti-platelet antibody. Seven mice from each group were sacrificed 1 and 2 weeks after induction, respectively. Ten mice from each group were sacrificed 5 weeks after induction. One mouse in the PD group died of internal bleeding at 1 week after induction. **(B)** Kinetic changes in mean bodyweight. **(C)** Kinetic changes in mean lesion weight. **(D)** Kinetic changes in mean hotplate latency. The values show the means ± SDs. ^#^0.05 < p = 0.053; *p < 0.05; NS: not statistically significant (p > 0.05) for the difference between the testing group and the Control group (by Wilcoxon’s test). PD, platelet depletion; Ab, antibody.

For Experiment 1, 48 mice were randomly divided into two equal-sized groups: the isotype control (IC) group and the platelet depletion (PD) group. For platelet depletion, mice were injected intravenously with an anti-platelet antibody (R300, Emfret Analytics, Eibelstadt, Germany), and for the IC group, the mock isotype antibody matched with anti-platelet antibody (rat IgG1) (C301, Emfret Analytics), both at a dosage of 2 μg/g bodyweight for each injection. For both groups, the real or mock antibody was injected at the day of the induction of endometriosis (day 0), and then every 5 days consecutively, with the last injection being given on day 30. The schematic depiction of the experimental design is shown in **Figure 1A**. As reported previously by our group, the average platelet count in mice injected with the identical anti-platelet antibody (R300) at a 5-day interval was 15 × 10^9^/L in the blood, while those injected with the matched IC had an average platelet count of 136 × 10^9^/L. That is, the platelet count was reduced by 89.0% (5). At the end of the first and second weeks after induction of endometriosis, seven mice each were sacrificed; at the end of the fifth week after induction, 10 mice each were sacrificed. Before the induction of endometriosis and before sacrifice, the body weight was measured and the hotplate latency test was administered to all mice.

For Experiment 2, 32 mice were randomly divided into four equal-sized groups: the Control (CT) group, the platelet depletion (PD) group, the Tregs depletion (TD) group, and the joint platelet and Tregs depletion (JD) group. For PD mice, mice were injected intravenously with anti-platelet antibody or the mock isotype antibody mentioned above (both from Emfret Analytics), at a dosage of 2 μg/g bodyweight for each injection, and at seven different time points for the four groups. For TD mice, mice were injected intraperitoneally with monoclonal anti-CD25 antibody (PC61) or the mock isotype antibody matched with PC61 (rat IgG1) (both from Bio X Cell, West Lebanon, NH, USA), at a dosage of 10 μg/g bodyweight for each injection, and at six different time points for the four groups. For CT mice, the mock antibody matching with the anti-platelet antibody was first injected on the day for the induction of endometriosis (day 0), and then every 5 days consecutively, with the last injection being given on day 30, and the mock antibody matching with PC61 was injected at 3 days before, 3 days after the induction of endometriosis, and then every 7 days consecutively, with the last injection being given on day 32. For the JD mice, the anti-platelet antibody was given at all seven time points that were identical to the CT group; PC61 was given at all six time points that were identical to the CT group. For the PD or TD group, anti-platelet antibody or PC61, and the mock antibody for PC61 or anti-platelet antibody were injected at the time points identical to the CT group. Our pilot experiment using flow cytometry showed that, while the isotype IgG group had an average of 12.84% of CD25+ cells in CD4+ cells, the same percentage reached a low level of 0.86% (a reduction of 93.3%) at Day 3 after injection of PC61, and remained below 1% (over 92.2% reduction) at Day 10. The trend was very similar in the percentage of FOXP3+ cells in CD4+ cells, reducing the percentage from 6.04% on Day 0 of injection to 0.64% (a reduction of 89.4%) on Day 3 after injection and remained below 1% (over 83.4% reduction) on Day 10 (data not shown). In light of this result and to ensure a low level of Tregs in blood, we injected PC61 first at 3 days before the induction of endometriosis, and then at a 7-day interval after the induction. All mice were sacrificed 35 days after induction. Before the induction of endometriosis and before sacrifice, the body weight was measured and the hotplate test was administered to all mice.

### Hotplate Test Procedure and Lesion Measurement

Given that women with endometriosis and rodents with induced endometriosis exhibit central sensitization, and since mice are not vocal about their pain severity, we measured the hotplate latency of mice as a proxy. A commercially available Hot Plate Analgesia Meter (Model BME-480, Institute of Biomedical Engineering, Chinese Academy of Medical Sciences, Tianjin, China) was used to perform the hotplate test, as reported previously (37, 38).

The endometriotic lesions were carefully excised and weighed and then fixed for immunohistochemistry (IHC) analysis or Masson Trichrome Staining. The dry weight of lesions was determined as previously reported (39).

### Immunohistochemistry

Tissue samples were fixed with 4% paraformaldehyde (w/v) and embedded with paraffin. Serial sections of 4 μm were obtained from each block, with one slide being stained for hematoxylin and eosin to confirm the pathological diagnosis, and the other slides stained for TGF-β1, glycoprotein A repetitions predominant (GARP), vimentin, E-cadherin, α-smooth muscle actin (α-SMA), collagen-I, FOXP3, GATA-binding protein 3 (GATA-3), T-box expressed in T cells (T-bet), RAR-related orphan receptor gamma T (RORγT), CD68, CD163, inducible nitric oxide synthase (iNOS), CD41, p-Smad3, p-PDGFR-β, p-Akt, and PDGF-BB. For positive controls, human liver tissue samples were used for staining of TSLP following the product instruction; mouse spleen tissue samples were used for staining of PDGF-BB (40), FOXP3 (41), T-bet (42), GATA-3, and RORγT (43), and for p-PDGFR-β following the product instruction. CD163, iNOS, CD41, and α-SMA are known to be expressed in mouse endometriotic lesions (8), hence their staining in mouse endometriosis tissue samples were served as positive control in themselves. Human breast cancer tissue samples were used for staining of GARP (44), ovarian endometrioma tissue samples were used for CD68 (45), p-Smad3 (9), and p-Akt (46), deep endometriosis tissue samples were used for TGF-β1, normal endometrium tissue samples were used for E-cadherin (9), and human adenomyosis tissue samples were used for vimentin (47). For negative controls, tissue samples were incubated with rabbit serum (for p-PDGFR-β, p-Akt, TSLP, PDGF-BB, T-bet, GATA-3, RORγT, CD163, GARP, FOXP3, vimentin, α-SMA, iNOS, CD41, TGF-β1, p-Smad3) or mouse serum (for CD68, E-cadherin) instead of primary antibodies.

Routine deparaffinization and rehydration procedures were performed. For antigen retrieval, the slides were heated at 98°C for 20 min in an EDTA buffer (pH 8.0; Shanghai Sun BioTech Co, Shanghai, China) for staining of T-bet, GATA-3, RORγT, CD163, p-Smad3, p-PDGFR-β, and p-Akt; or for 30 min in citrate buffer (pH 6.0) for staining of GARP, FOXP3, CD68, iNOS, CD41, α-SMA, vimentin, E-cadherin, PDGF-BB, and TSLP, and then cooled naturally to room temperature. **Table 1** lists the names, along with catalog numbers, of the primary antibodies used in this study and their respective diluted concentrations.

**Table 1:** List of names and catalog numbers of all antibodies used in immunohistochemistry in this study.

Antibody name	Catalog number	Vendor name and location	Concentration
CD68	Ab955	Abcam, Cambridge, UK	1:200
CD163	Ab182422	Abcam	1:500
TGF-β1	Ab215715	Abcam	1:100
GARP	Orb36818	Biorbyt, Cambridge, UK	1:25
E-cadherin	Ab231303	Abcam	1:400
Vimentin	Ab45939	Abcam	1:700
α-SMA	Ab5694	Abcam	1:100
FOXP3	Ab54501	Abcam	1:1,200
T-bet	Ab91109	Abcam	1:100
GATA-3	Ab199428	Abcam	1:500
RORγT	Orb185932	Biorbyt	1:200
iNOS	Ab115819	Abcam	1:100
CD41	Ab134131	Abcam	1:100
p-Smad3	Ab52903	Abcam	1:100
p-PDGFR-β	Ab62437	Abcam	1:50
p-Akt	4060	CST, Boston, MA, USA	1:50
PDGF-BB	Ab23914	Abcam	1:500
TSLP	Ab188766	Abcam	1:200

TGF-β1, transforming growth factor-β1; GARP, glycoprotein A repetitions predominant; α-SMA, alpha-smooth muscle actin; T-bet, T-box expressed in T cells; GATA-3, GATA-binding protein 3; RORγT, RAR-related orphan receptor gamma T; iNOS, inducible nitric oxide synthase; p-Smad3, phosphorylated Smad3; p-PDGFR-β, phosphorylated platelet-derived growth factor receptor-β; p-Akt, phosphorylated Akt; PDGF-BB, platelet-derived growth factor-BB; TSLP, thymic stromal lymphopoietin.

The slides were incubated with the primary antibodies overnight at 4°C. The next day, slides were incubated with horseradish peroxidase (HRP) labeled secondary antibody detection reagent (Sunpoly-HII; BioSun Technology Co., Ltd., Shanghai, China) at room temperature for 30 min. The bound antibody complexes were stained with diaminobenzidine for 1–2 min or until noticeable by microscopic examination, and then counterstained with hematoxylin for 2 min and mounted. Images were taken with a microscope (Olympus BX51; Olympus, Tokyo, Japan) fitted with a digital camera (Olympus DP70; Olympus). The density of FOXP3, T-bet, RORγT, GATA-3, CD68, CD163, iNOS, and CD41 positive cells in the acquired images were counted using Image Pro-Plus 6.0 (Media Cybernetics, Inc, Bethesda, MD, USA) software. Positive cells were automatically counted using Image Pro-Plus 6.0 by establishing a color density threshold to distinguish positive cells. For other immune-stained markers, five randomly selected images at ×400 magnifications of each sample were taken to obtain a mean optional density value with Image Pro-Plus 6.0 as reported previously (48).

### Masson Trichrome Staining

Masson Trichrome staining was used for the detection of collagen fibers in tissue samples. Tissue slides were deparaffinized in xylene and rehydrated in a graded alcohol series and then immersed in the Bouin solution, which was made with 75 ml of saturated picric acid, 25 ml of 10% formalin (w/v) solution, and 5 ml of acetic acid, at 37°C for 2 h. Tissue slides were stained with the Masson Trichrome Staining Kit (Baso, Wuhan, China) following the manufacturer’s instructions. The areas of the blue-stained collagen fiber layer in proportion to the entire field of the ectopic implants were calculated by the Image Pro-Plus 6.0.

### Statistical Analysis

The comparison of distributions of continuous variables between or among two or more groups was made with the Wilcoxon’s test or Kruskal’s test, respectively. Pearson’s or Spearman’s rank correlation coefficient was used for evaluating correlations between two variables when at least one is ordinal or when both were continuous. To use data structurally and more efficiently, multiple linear regression was used to evaluate the effect of platelet and/or Tregs depletion and duration of induction on IHC parameters, or bodyweight or hotplate latency. P values < 0.05 were considered statistically significant. All computations were made with R 4.0.2 (www.r-project.org).

## Results

### Depletion of Platelets Retards the Progression of Endometriosis

We first investigated the effect of platelet depletion on the development and fibrogenesis of endometriosis in mice based on a serial experimentation. Starting 2 days before induction, the platelet-depleting antibody was injected every 5 days during the development of endometriosis to ensure that platelets were kept to a minimum while the control mice received injection of an isotype-matched (mock) antibody (**Figure 1A**). The identical platelet depletion procedure was found to yield a reduction of platelets by 89.0% (5). Seven mice each from IC (isotype control) and PD (platelet depletion) groups were sacrificed at 1 and 2 weeks after induction, and then 10 mice each were sacrificed from the two groups 5 weeks after induction (**Figure 1A**). One mouse in the PD 1-week group died of internal bleeding at 1 week after induction.

No differences were detected in bodyweight between the two groups before, and 1, 2, and 5 weeks after the induction of endometriosis (all p-values ≥ 0.10; **Figure 1B**). Multiple linear regression incorporating time of measurement and group identity (PD *vs.* IC) indicated that there is no difference between the two groups, but the bodyweight progressively increased over time (p < 2.2 × 10^−16^, *R^2^* = 0.82).

We found that platelet depletion resulted in nearly significantly reduced lesion weight at 1 and 2 weeks after induction but significantly at 5^th^ week (p = 0.051, p = 0.053, and p = 0.017, respectively; **Figure 1C**). However, multiple linear regression incorporating the time of measurement and group identity (PD *vs.* IC) indicated that PD was indeed associated with significantly lower lesion weight (p = 1.2 × 10^−4^) while longer duration of induction was associated with significantly increased lesion weight (p = 4.3 × 10^−9^, *R^2^* = 0.61; **Figure 1C**).

As expected, there was no significant difference in baseline hotplate latency between the two groups (p = 0.88; **Figure 1C**). While the PD mice had slightly decreased latency 1 and 2 weeks after the induction, the latency in IC mice appeared to decrease more rapidly, and this trend appeared to continue as time elapsed (**Figure 1D**). However, no significant difference in latency between the two groups was found at 1 and 2 weeks (p = 0.28 and p = 0.12, respectively; **Figure 1C**). Five weeks after the induction, the difference was nearly significant (p = 0.053; **Figure 1D**). Multiple linear regression incorporating time of measurement and group identity indicated that PD was associated with significantly positive change (p = 0.034) while longer duration of induction significantly decreased latency (p = 6.9 × 10^−5^, *R^2^* = 0.21; **Figure 1D**). Indeed, while at the 5^th^ week after induction the IC mice had significantly reduced hotplate latency as compared with the baseline levels (p = 0.013), no such reduction was found in PD mice (p = 0.15).

### Platelet Depletion Disrupts a Predominantly Type 2 Immunity in Lesional Microenvironment and Stalls Lesional Fibrogenesis

We next performed immunohistochemistry analyses for markers of platelets (CD41+), macrophages (CD68+), M1 (iNOS+), and M2 (CD163+) macrophages as well as TSLP staining for endometriotic lesions.

CD41 staining was seen in the membrane of platelets. CD68 staining was seen in the membrane of macrophages, while CD163 staining was seen in the membrane and cytoplasm of putative M2 macrophages. The staining of iNOS was seen in the cytoplasm of putative M1 macrophages and endometriotic epithelial cells, but the latter could be distinguished by cell morphology. As such, iNOS+ epithelial cells were not included in the counting, and the scoring was done only for macrophages. Also, TSLP staining was detected diffusely in the stromal area and the epithelial cells of endometriotic lesions.

**Figure 2:**
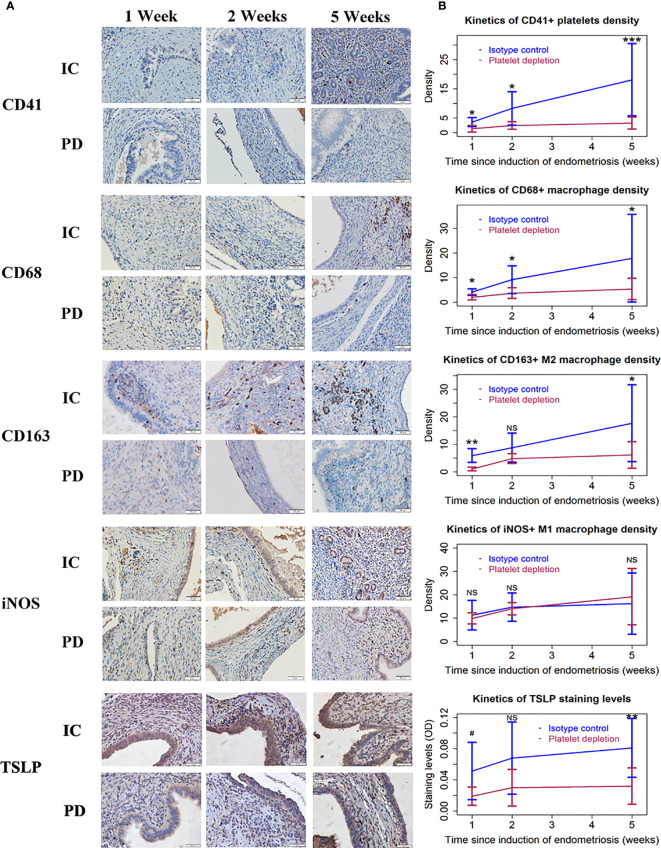
Platelets, macrophages, and TSLP expression in endometriotic lesions of the two groups of mice in Experiment 1. **(A)** Representative immunohistochemical staining of CD41, CD68, CD163, iNOS, and TSLP staining in the two groups. All magnifications were ×400. Scale bar = 50 µm. **(B)** Kinetic changes in the density of CD41+ (platelet), CD68+ (macrophage), CD163+ (M2), and iNOS+ (M1) cells, and the staining level of TSLP. The values show the means ± SDs. 0.05<#<0.1 *p < 0.05; **p < 0.01; ***p < 0.001; NS, not statistically significant (p > 0.05) for the difference between the testing group and the Control group (by Wilcoxon’s test). n = 7 for each group in 1 and 2 weeks; n = 10 for each group in 5 weeks. One mouse in the PD group died of internal bleeding at 1 week after induction. IC, isotype control; PD, platelet depletion; TSLP, thymic stromal lymphopoietin.

We found that the density of activated platelets was progressively increased in endometriotic lesions in IC mice (**Figure 2A**; **Table 2**). In addition, the density of macrophages (CD68+) and M2 macrophages (CD163+) was both elevated as endometriotic lesions progressed (**Figure 2** and **Table 2**). In contrast, PD resulted in a significantly lower density of platelets, macrophages, and M2 macrophages (**Figure 2** and **Table 2**). Interestingly, the density of M1 macrophages (iNOS+) did not change much during the entire period of lesional development, nor was there any significant difference between the two groups of mice (all p-values ≥ 0.63; **Figure 2** and **Table 2**). Consistent with these changes, the overall lesional staining of TSLP increased slightly but significantly increased during the entire experimental period (p = 0.049), but was significantly reduced in mice with PD (p = 1.6 × 10^−5^, *R^2^* = 0.38; **Figure 2** and **Table 2**). These results indicate that platelets can promote the aggregation of M2 macrophages in lesions and M2 macrophages may facilitate the progression of endometriosis.

**Table 2:** Results of multiple linear regression analysis of immunohistochemistry and histochemistry data from Experiment 1.

Marker	Effect of duration of induction	Effect of platelet depletion	*R^2^* value
CD41+ platelets	↑↑↑	↓↓↓	0.54
CD68+ macrophages	↑↑↑	↓↓↓	0.36
CD163+ macrophages	↑↑↑	↓↓↓	0.41
iNOS+ macrophages	—	—	0.09
TSLP	↑	↓↓↓	0.38
FOXP3+ Tregs	↑↑	↓↓↓	0.50
T-bet+ Th1 cells	↑↑↑	↓↓	0.33
GATA-3+ Th2 cells	↑↑↑	↓↓↓	0.61
RORγT+ Th17 cells	—	↓↓↓	0.26
GARP	↑↑↑	↓↓↓	0.78
Extent of lesional fibrosis	↑↑↑	↓↓↓	0.44

The symbol “↑” denotes elevation while “↓” denotes reduction. The number of “↑” or “↓” indicates the statistical significance level associated with variable, in which one, two, and three such symbols indicate that the p-value is <0.05, 0.01, and 0.001, respectively. The symbol “—” means no association.

**Figure 3:**
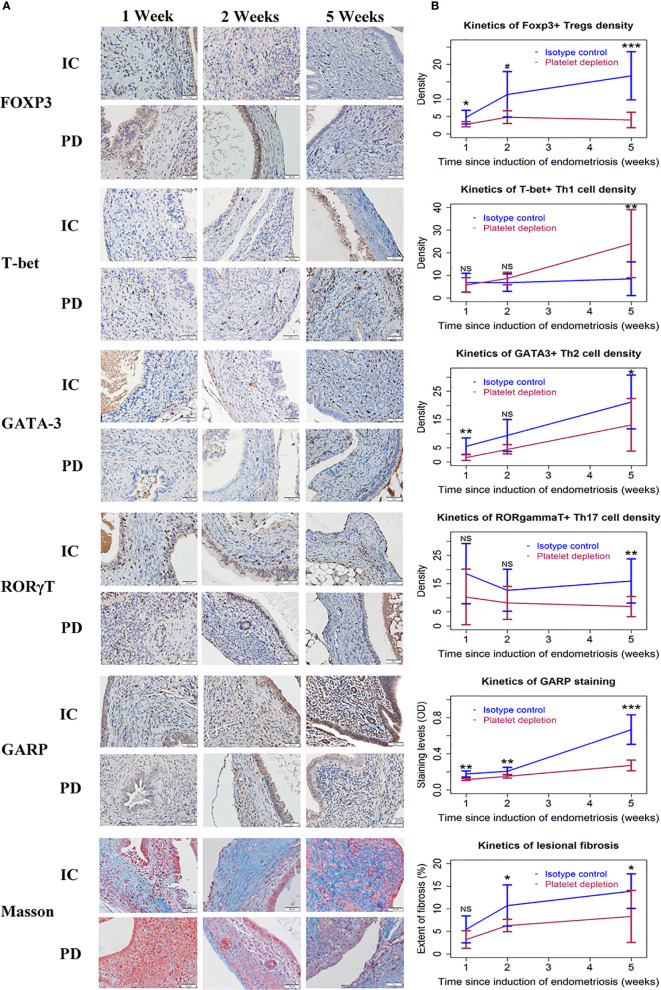
The distribution of T cells, GARP staining, and Masson trichrome staining in endometriotic lesions of the two groups of mice in Experiment 1. **(A)** Representative immunohistochemical staining of FOXP3, T-bet, GATA-3, RORγT, GARP, along with Masson trichrome staining in the two groups. All magnifications were ×400. Scale bar = 50 µm. **(B)** Kinetic changes in the density of FOXP3+ (Treg), T-bet+ (Th1), GATA-3+ (Th2), and RORγT+ (Th17) cells, the staining level of GARP, and the percentage of fibrotic content in the two groups. The values show the means ± SDs. *p < 0.05; **p < 0.01; ***p < 0.001; NS, not statistically significant (p > 0.05) for the difference between the testing group and the Control group (by Wilcoxon’s test). n = 7 for each group in 1 and 2 weeks; n = 10 for each group in 5 weeks. One mouse in the PD group died of internal bleeding at 1 week after induction. IC, isotype control; PD, platelet depletion; GARP, glycoprotein A repetitions predominant.

We further stained markers of Tregs, Th1, Th2, and Th17, and GARP, and quantified the extent of lesional fibrosis by Masson trichrome staining. FOXP3, T-bet, GATA-3, and RORγT were stained positive in the nuclei of Tregs, Th1, Th2, and Th17, respectively. GARP is a transmembrane protein retained at the surface of Tregs and a few other cell types (44, 49), and is involved in TGF-β1 activation by Tregs (50, 51). GARP staining was seen in the membrane of activated platelets, Tregs, and epithelial cells of endometriotic lesions (**Figure 3A**).

We found that, as lesions progressed, the density of both Tregs and Th2 cells increased precipitously, but that of Th1 cells remained mostly unchanged (**Figure 3** and **Table 2**). However, platelet depletion resulted in significantly reduced density of Tregs and Th2 cells while progressively increased the density of Th1 cells in lesions (**Figure 3** and **Table 2**). In fact, while the density of Tregs in 5-week old lesions from IC mice was significantly higher than that in 1-week old ones (p = 0.0002), no such significant difference in PD lesions was seen (p = 0.25; **Figure 3**). Conversely, while the density of Th1 cells in 5-week old lesions from IC mice was essentially unchanged as compared with that in 1-week old ones (p = 0.73), in PD mice the density in 5-week old lesions was significantly higher than that in 1-week old ones (p = 0.0014; **Figure 3**).

In contrast to the increasing density of Tregs and Th2 cells and also to somewhat constantly low density of Th1 cells in IC mice, the density of Th17 cells (RORγT+) appeared to be relatively unchanged as lesions progressed, but at higher levels (**Figure 3**). The density also appeared to be relatively constant in PD mice, but it was significantly lower than that of IC mice (**Figure 3** and **Table 2**). In fact, the density of Th17 cells in 5-week old lesions from both IC and PD mice was similar to that in 1-week old ones (p = 0.74 and p = 1.0, respectively; **Figure 3** and **Table 2**).

Consistently, the lesional staining of GARP in IC mice increased progressively as lesions aged, but PD significantly reduced the density (**Figure 3** and **Table 2**). In addition, the extent of lesional fibrosis also increased progressively, but PD significantly lowered it (**Figure 3** and **Table 2**).

Taken together, we can conclude that platelets promoted the increasing aggregation of Tregs and Th2 cells as well as M2 macrophages along with a constant low-level aggregation of Th1 cells during the progression of endometriosis, which facilitated the expression of TSLP and GARP and likely induced TGF-β1 activation, resulting in lesional progression and fibrogenesis. Platelet depletion, however, facilitated the aggregation of Th1 cells while suppressed the aggregation of Tregs, Th2, and Th17 cells as well as M2 macrophages, yielding retarded lesional progression and stalled lesional fibrogenesis.

**Figure 4:**
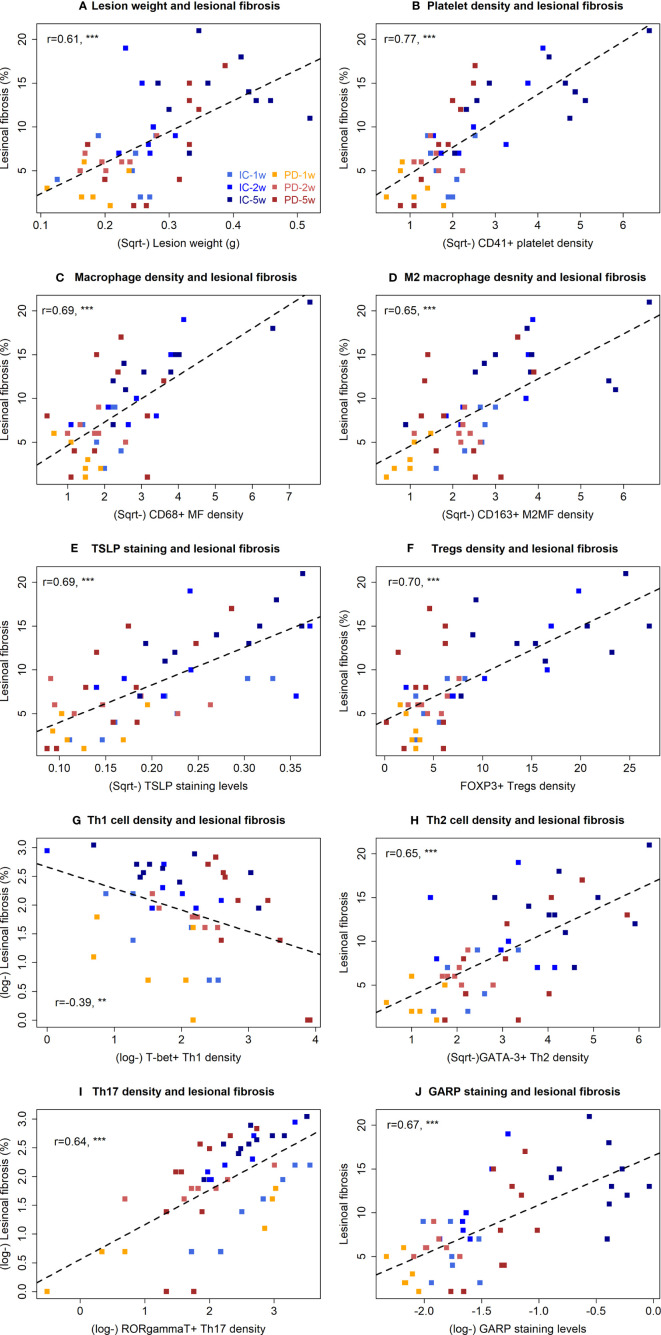
Correlation between lesion weight, or immunohistochemistry staining and fibrotic proportion of endometriotic lesions in the two groups of mice in Experiment 1. Scatter plots showing the relationship between the extent of lesional fibrosis and the lesion weight **(A)**, the density of platelets **(B)**, macrophages **(C)**, M2 macrophages **(D)**, Tregs **(E)**, Th1 cells **(F)**, Th2 cells **(G)**, and Th17 cells **(H)**, or the staining levels of TSLP **(I)** and GARP **(J)**. Each dot represents one data point from one patient. The dashed line is the regression line. The number in each figure is the Pearson correlation coefficient, followed by a symbol indicating the statistical significance level. **p < 0.01; ***p < 0.001. W, week; PD, platelet depletion; MF, macrophages; GARP, glycoprotein A repetitions predominant; TSLP, thymic stromal lymphopoietin; Sqrt, square root.

The extent of lesional fibrosis was positively correlated with the lesion weight, the density of platelets, infiltrating macrophages, M2 macrophages, Tregs, Th2 and Th17 cells, and lesional staining levels of TSLP and GARP, but negatively with the density of Th1 cells (**Figure 4**). Taken together, these data suggest that through promoting a predominantly type 2 immunity in lesions, promoting the aggregation of Tregs, Th2 cells, and M2 macrophages but keeping a low-level of Th1 cell aggregation in endometriotic lesions and possibly activating the TGF-β1/Smad3 signaling pathway, the platelets promote lesional fibrogenesis in endometriosis.

### Depletion of Platelets and/or Tregs Retards the Progression of Endometriosis

**Figure 5:**
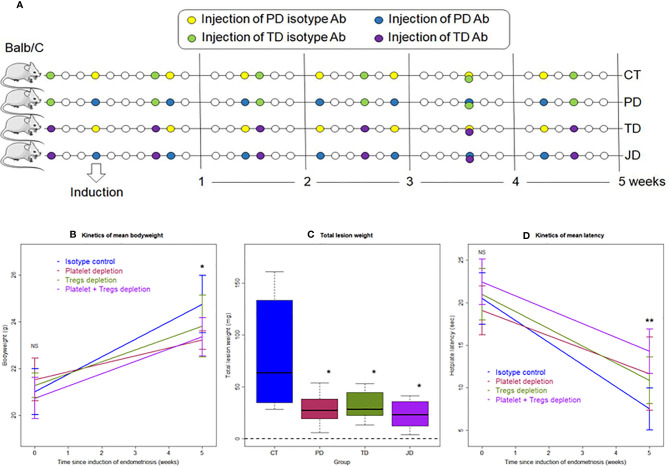
Schematic illustration of the experimental design, kinetics of bodyweight, lesion weight, and hotplate latency of the four groups of mice in Experiment 2. **(A)** Schematic illustration of the experimental design. The yellow circles represent the injection of mock isotype antibody for the anti-platelet antibody; the blue circles represent the injection of anti-platelet antibody; the purple circles represent the injection of anti-CD25 antibody (PC61); and the green circles represent the injection of mock isotype antibody for PC61. All mice were sacrificed 35 days after induction. **(B)** Kinetic changes in mean bodyweight. **(C)** Boxplots showing the Lesion weight. **(D)** Kinetic changes in mean hotplate latency. The values show the means ± SDs. *p < 0.05; **p < 0.01; NS: not statistically significant (p > 0.05) for the difference between the testing group and the Control group (by Wilcoxon’s test). n = 8 for each group. CT, Control group; PD, platelet depletion; TD, Tregs depletion; JD, joint (simultaneous) depletion of platelet and Treg; Ab, antibody.

In view of the findings from Experiment 1 that platelet depletion retarded the progression of endometriosis possibly through the disruption of the type 2 immunity in lesional microenvironment, we next carried out Experiment 2 to further explore the possible interactions between platelets and Tregs in the progression and fibrogenesis of endometriosis (**Figure 5A**). Thirty-two Balb/c mice were randomly divided into four groups: the PD group, which was injected with platelet depletion antibody; the TD group, which was injected with Tregs depletion antibody; the JD group, which was injected with antibodies for both platelet and Tregs depletion (joint depletion); and, finally, the CT group, which was injected with isotype mock antibody. Five weeks after the induction of endometriosis, all mice were sacrificed (**Figure 5A**). In our pilot experiment using flow cytometry, we found that the percentage of of CD25+ cells in CD4+ cells was reduced by 93.3% at Day 3 after injection of PC61 and the reduction remained to be over 92.2% at Day 10 after. The trend was very similar in the percentage of FOXP3+ cells in CD4+ cells, yielding a reduction from 6.04% on Day 0 of injection to 0.64% (a reduction of 89.4%) on Day 3 after injection and remained below 1% (over 83.4% reduction) on Day 10 (data not shown). In light of this result and to ensure a low level of Tregs in blood, we injected PC61 first at 3 days before the induction of endometriosis, and then at a 7-day interval after the induction. During the whole experiment, no mice died.

While no difference in bodyweight was found at the beginning of the experiment (p = 0.33), at the end of the experiment there was a significant difference among the four groups of mice (p = 0.023; **Figure 5B**). Multiple linear regression incorporating the time of measurement, whether received platelet depletion, or Tregs depletion indicated that the bodyweight overall was progressively increased over time (p = 5.2 × 10^−16^) and platelet, but not Tregs, depletion resulted in reduced bodyweight (p = 0.048; *R^2^* = 0.67; **Figure 5B**). The reduced bodyweight might be due to possible internal bleeding because of platelet depletion.

Compared with the CT mice, the lesion weight in PD, TD, and JD groups was reduced, on average, by 64.8, 60.5, and 71.0%, respectively (all p-values ≤ 0.036; **Figure 5C**). Multiple linear regression incorporating the dummy variables indicating as whether the mouse received platelet depletion, or Tregs depletion indicated that platelet depletion and Tregs depletion significantly reduced the lesion weight (p = 0.0013, and p = 0.0023, respectively), but the joint depletion did not further reduce the lesion weight (p = 0.043; *R^2^* = 0.42; **Figure 5C**).

While there was no difference in hotplate latency at the baseline among the four groups (p = 0.23), by the end of experiment there was a significant difference in latency among them (p = 0.0058; **Figure 5D**). Multiple linear regression incorporating the time of measurement, and the dummy variables indicating as whether received platelet depletion, or Tregs depletion indicated that while the time elapsed since induction significantly reduced the latency (p < 2.2 × 10^−16^), both platelet depletion and Tregs depletion significantly prolonged the latency (p = 0.017, and p = 0.0026, respectively; *R^2^* = 0.74; **Figure 5D**).

### Platelets and/or Tregs Depletion Results in Reduced Aggregation of Immune Cells Suggestive of Type 2 Immunity in Endometriotic Lesions and Stalled Lesional Progression and Fibrogenesis

**Figure 6:**
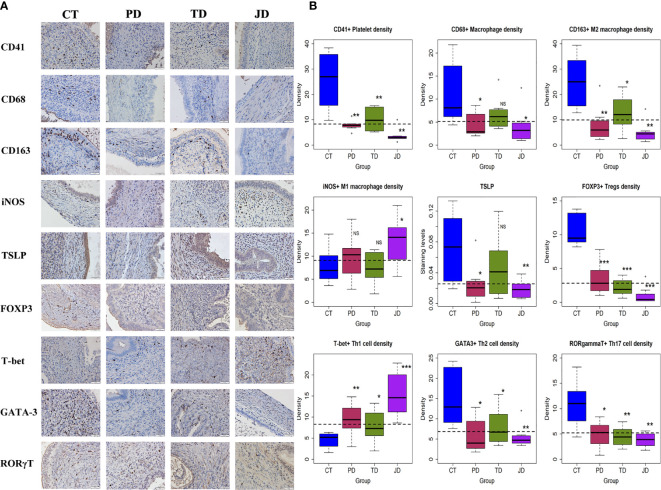
Platelets, macrophage subsets, and T cell subsets in endometriotic lesions of the four groups of mice in Experiment 2. **(A)** Representative immunohistochemical staining of CD41, CD68, CD163, iNOS, TSLP, FOXP3, T-bet, GATA-3, and RORγT in the four groups. All magnifications were ×400. Scale bar = 50 µm. **(B)** Boxplots showing the density of CD41+ (platelet), CD68+ (macrophage), CD163+ (M2), and iNOS+ (M1), FOXP3+ (Treg), T-bet+ (Th1), GATA-3+ (Th2), RORγT+(Th17) cells, and the staining level of TSLP. *p < 0.05; **p < 0.01; ***p < 0.001; NS, not statistically significant (p > 0.05) for the difference between the testing group and the Control group (by Wilcoxon’s test). n = 8 for each group. CT, Control group; PD, platelet depletion; TD, Treg depletion; JD, joint (simultaneous) depletion of platelet and Treg; TSLP, thymic stromal lymphopoietin.

We then evaluated the density of platelets (CD41+), macrophages (CD68+), M2 (CD163+) and M1 (iNOS+) macrophages, Tregs (FOXP3+), Th1 (T-bet+), Th2 (GATA-3+), and Th17 (RORγT+), along with TSLP staining in endometriotic lesions by IHC (**Figure 6A**). As expected, the lesional platelet density was significantly reduced in PD and JD mice as compared with the CT group (both p-values = 0.0013; **Figure 6B**, **Table 3**), so was in TD mice (p = 0.006). PD and JD mice also had significantly reduced macrophage density in lesions (p = 0.024 and p = 0.012, respectively; **Figure 6B**, **Table 3**), but not in TD mice (p = 0.10). While all treatment groups of mice had significantly reduced the lesional density of M2 macrophages (p = 0.0011, p = 0.028, and p = 0.0019, respectively; **Figure 6B**, **Table 3**), only the JD mice had significantly elevated density of M1 macrophages (p = 0.038; **Figure 6B**, **Table 3**). In JD mice, the density of platelets and M2 macrophages was further reduced as compared with TD mice (both p-values ≤ 0.038; **Figure 6**), while the density of M1 macrophages did not change significantly compared with the TD group (p = 0.052; **Figure 6B**, **Table 3**), suggesting that platelets and Tregs may have an additive effect on the promotion of M2 dominance over M1 in endometriotic lesions. Platelet depletion, but not Tregs depletion alone, significantly reduced the lesional staining levels of TSLP (p = 0.021, and p = 0.0047, respectively; **Figure 6B**, **Table 3**), suggesting that platelets, but not Tregs, are capable of promoting the expression of TSLP, which is likely to facilitate the ensuing type 2 immunity predominance in endometriotic lesions.

**Table 3:** Results of multiple linear regression analysis of immunohistochemistry and histochemistry data from Experiment 2.

Marker	Effect of platelet depletion	Effect of Tregs depletion	Effect of Joint platelet and Tregs depletion	*R^2^* value
CD41+ platelets	↓↓↓	↓↓↓		0.70
CD68+ macrophages	↓↓	—	—	0.30
CD163+ macrophages	↓↓↓	↓↓	—	0.54
iNOS+ macrophages	—	—	—	0.13
TSLP	↓↓	—	—	0.26
FOXP3+ Tregs	↓↓↓	↓↓↓	↑	0.77
T-bet+ Th1 cells	↓↓	↓↓	—	0.42
GATA-3+ Th2 cells	↓↓↓	↓	↑	0.41
RORγT+ Th17 cells	↓↓↓	↓↓↓	↑	0.54
TGF-β1	↓↓↓	↓↓↓	↑	0.70
GARP	↓↓↓	↓↓	—	0.51
p-Smad3	↓↓↓	↓↓↓	↑↑↑	0.83
p-PDGFR-β	↓↓↓	↓↓↓	↑↑	0.74
PDGF-BB	↓↓↓	↓↓↓	↑	0.73
p-Akt	↓↓↓	↓↓↓	↑↑	0.77
E-cadherin	↓↓↓	↓	—	0.52
Vimentin	↓↓↓	↓↓	—	0.47
α-SMA	↓↓↓	↓↓↓	↑↑	0.67
Extent of lesional fibrosis	↓↓↓	↓↓↓	↑↑	0.76

The symbol “↑” denotes elevation while “↓” denotes reduction. The number of “↑” or “↓” indicates the statistical significance level associated with variable, in which one, two, and three such symbols indicate that the p-value is <0.05, 0.01, and 0.001, respectively. The symbol “—” means no association.

In the three treatment groups, the lesional density of Tregs, Th2, and Th17 was significantly lower, and the density of Th1 was significantly higher, than the CT group (all p-values ≤ 0.046; **Figure 6B**, **Table 3**). This suggests that both platelets and Tregs promoted, first, the aggregation of Tregs as well as Th2 and Th17 cells, but suppressed the aggregation of Th1 cells in endometriotic lesions. Of particular interest, the density of Tregs in the JD mice was significantly lower than that of PD and TD mice (both p-values ≤ 0.030; **Figure 6B**; **Table 3**), suggesting that platelet depletion further facilitated the reduction in Tregs density in lesions. The density of Th1 in the JD mice was significantly higher than either PD or TD mice (both p-values ≤ 0.036; **Figure 6B**, **Table 3**), indicating that platelets and Tregs have an additive suppressive effect on Th1 aggregation, which can also be seen from the lack of significant interaction (**Table 3**). When platelets and Tregs were depleted jointly, there was no significant difference in Th17 density between the JD group and the PD or TD group (both p-values ≥ 0.25; **Figure 6B**, **Table 3**), suggesting that both platelets and Tregs facilitated Th17 aggregation *via* the same pathway (as seen by the positive sign of the regression coefficient, which was significant).

### Evidence for the Activation of the TGF-β1/Smad3 and PDGFR-β/PI3K/Akt Signaling Pathways by Platelets and Tregs

**Figure 7:**
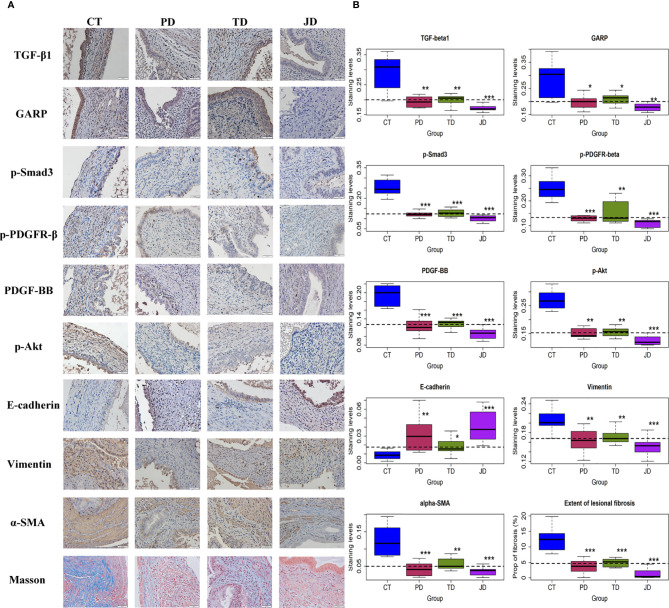
Markers of the TGF-β and PDGF signaling pathways and fibrosis in endometriotic lesions of the four groups of mice in Experiment 2. **(A)** Representative immunohistochemical staining and Masson trichrome staining in the four groups. All magnifications were ×400. Scale bar = 50 µm. **(B)** Boxplots showing the summary of immunohistochemistry analyses of GARP, E-cadherin, vimentin, α-SMA, TGF-β1, p-Smad3, p-PDGFR-β, PDGF-BB, p-Akt, and the percentage of fibrotic content in endometriotic lesions evaluated by Masson trichrome staining. *p < 0.05; **p < 0.01; ***p < 0.001 for the difference between the testing group and the Control group (by Wilcoxon’s test). n = 8 for each group. CT, Control group; PD, platelet depletion; TD, Treg depletion; JD, joint (simultaneous) depletion of platelet and Treg; TGF-β1, transforming growth factor-β1; GARP, glycoprotein A repetitions predominant; PDGF-BB, platelet-derived growth factor-BB; p-PDGFR-β, phosphorylated-platelet-derived growth factor receptor-β; α-SMA, α-smooth muscle actin.

Activated platelets and Tregs are known to release TGF-β1 and PDGF, two potent profibrotic cytokines (3, 52, 53). We thus set out to detect the lesional expression of TGF-β1/Smad3 and the markers of the PDGFR-β/PI3K/Akt signaling pathways by IHC. Platelets have been previously reported to activate the TGF-β1 and PDGF signaling pathways in endometriosis (3, 7). In our pilot experiment using real-time RT-PCR or Western blot, we also found that Tregs activated the TGF-β1 and PDGF signaling pathways in both endometriotic epithelial and stromal cells. The total proteins and phosphorylated forms of Smad3, PDGFR-β, and Akt were all evaluated by Western blot, but the changes were only found in the phosphorylated forms (data not shown). Consequently, we only evaluated the phosphorylated forms of these proteins in our IHC experiments. We found that TGF-β1 was stained positive mostly in the cytoplasm of ectopic endometrial epithelium and also in some stromal cells (**Figure 7A**). GARP staining was seen in the membrane of activated platelets, Tregs, and epithelial cells of endometriotic lesions (**Figure 7A**). The staining of p-Smad3 was seen prominently in the nuclei of epithelial cells and some stromal cells in the CT group, but its staining intensity was lower in the other three groups. Similarly, PDGF-BB staining was seen mostly in the cytoplasm of ectopic endometrial epithelium and also in some stromal cells in the Control mice, but its staining intensity was lower in the other three groups (**Figure 7**). The staining of p-PDGFR-β was seen in the membrane of stromal cells and some epithelial cells in controls, but less intense in the three treatment groups (**Figure 7A**). The staining of p-Akt was observed in the nucleus of stromal cells and some epithelial cells in the CT group, but less in the other three groups (**Figures 7A**).

After depletion of platelets or Tregs, the staining levels of TGF-β1, GARP, p-Smad3 and PDGF-BB, p-PDGFR-β, and p-Akt were significantly lower than the CT group (all p-values ≤ 0.028; **Figure 7B**, **Table 3**). While joint or simultaneous depletion of both platelets and Tregs also resulted in the reduction of the staining levels of these markers (all p-values ≤ 0.0006; **Figure 7B**), the significant and positive coefficients of the interaction term on the regression (**Table 3**) seem to indicate that platelets and Tregs impact on these signaling pathways more or less *via* identical pathways. Nonetheless, JD mice had significantly lower staining levels of TGF-β1, p-Smad3, PDGF-BB, p-PDGFR-β, and p-Akt than either PD or TD mice (all p-values < 0.05; **Figure 7B**). Similarly, the staining levels of GARP in JD mice was significantly lower than the TD, but not PD, mice (p = 0.012 and p = 0.10, respectively; **Figure 7**; **Table 3**), suggesting that platelet and Tregs may play an additive role in the activation of these two signaling pathways in endometriosis.

Due to the profibrotic propensity of both TGF-β1 and PDGF, we next evaluated the staining of markers of EMT and FMT by IHC, and the extent of lesional fibrosis by Masson trichrome staining. E-cadherin was mainly expressed in the cytoplasm and membrane of glandular epithelial cells, vimentin was expressed in the membrane of the stromal and some epithelial cells, and α-SMA was partially stained in the stroma of endometriotic lesions (**Figure 7A**). In Masson trichrome staining, the collagen fibers were stained blue (**Figure 7A**). Again, both PD and TD significantly elevated the staining levels of E-cadherin but significantly reduced staining levels of vimentin and α-SMA as well as the proportion of collagen fibers (all p-values ≤ 0.038; **Figure 7B**, **Table 3**), suggesting that depletion of either platelets or Tregs significantly suppressed the progression of EMT and FMT. The staining levels of E-cadherin in JD mice was significantly higher than the TD, but not PD, mice (p = 0.001, and p = 0.36, respectively; **Figure 7**, **Table 3**), while the staining levels of vimentin and α-SMA in JD mice was significantly lower than that the TD, but not PD, mice (both p-values ≤0.040, and both p-values >0.29; **Figure 7**, **Table 3**), suggesting that platelet depletion could further arrest EMT and FMT after Tregs depletion. Regardless, JD mice had significantly lower extent of lesional fibrosis than either PD or TD mice (both p-values <0.05; **Figure 7B**).

The extent of lesional fibrosis was positively correlated with the lesion weight, the density of platelets, macrophages, M2 macrophages, Tregs, Th17, and Th2 cells, but negatively correlated with the hotplate latency and the density of Th1 cells (**Table 4**). In addition, it correlated positively with the lesional staining levels of markers of the TGF-β and PDGF signaling pathways, such as TGF-β1, GARP, p-Smad3, PDGF-BB, p-PDGFR-β, and p-Akt, as well as the staining levels of TSLP. As expected, it correlated negatively with the staining levels of E-cadherin but correlated positively with lesional staining levels of vimentin and α-SMA (**Table 4**). Multiple linear regression incorporating lesion weight, platelet density, macrophage density, M2 macrophage density, Tregs density, Th2 and Th17 cell density, TGF-β1, p-Smad3, PDGF-BB, p-PDGFR-β, p-Akt, E-cadherin, vimentin, and α-SMA as covariates indicated that E-cadherin staining was negatively associated, while macrophage density, Tregs density, and p-Akt staining were positively associated, with the extent of lesional fibrosis (all p-values <0.011, *R^2^* = 0.94).

**Table 4:** Variables that are correlated with the extent of lesional fibrosis.

Variable name	Estimated correlation coefficient	p-value
Lesion weight	0.47	0.006
Hotplate latency at week 5	−0.45	0.009
Platelet density	0.86	2.8 × 10^−10^
Macrophage density	0.77	2.6 × 10^−7^
M2 macrophage density	0.76	4.1 × 10^−7^
Lesional TSLP staining levels	0.69	1.1 × 10^−5^
Tregs density	0.73	1.9 × 10^−6^
Th1 cell density	−0.81	2.1 × 10^−8^
Th2 cell density	0.72	3.3 × 10^−6^
Th17 cell density	0.70	9.6 × 10^−6^
Lesional TGF-β1 staining levels	0.85	1.1 × 10^−9^
Lesional GARP staining levels	0.83	3.6 × 10^−9^
Lesional p-Smad3 staining levels	0.88	5.0 × 10^−11^
Lesional p-PDGFR-β staining levels	0.85	2.9 × 10^−10^
Lesional PDGF-BB staining levels	0.85	6.3 × 10^−10^
Lesional p-Akt staining levels	0.86	1.8 × 10^−10^
Lesional E-cadherin staining levels	−0.78	1.2 × 10^−7^
Lesional vimentin staining levels	0.73	1.3 × 10^−6^
Lesional α-SMA staining levels	0.77	2.3 × 10^−7^

We also carried out a linear regression analysis of TGF-β1 staining levels using lesion weight, and densities of platelets, macrophages, M2 macrophages, Tregs, Th2, and Th17 cells as covariables. We found that the platelet density and Tregs density in lesions were the only two variables that are correlated positively with TGF-β1 staining levels (both p-values <3.2 × 10^−5^, *R^2^* = 0.83). Similarly, linear regression analysis of PDGF-BB staining levels on the same set of covariables identified lesion weight and platelet density as the two variables that are positively associated with the staining levels (both p-values < 0.004, *R^2^* = 0.88).

## Discussion

Through two mouse experiments, we have shown that, by disrupting predominantly a type 2 immune response in lesional microenvironment, both platelets and Tregs depletion decelerated lesional progression and fibrogenesis, likely through the suppression of the TGF-β1/Smad3 and PDGFR-β/PI3K/Akt signaling pathways. In particular, platelet depletion resulted in significantly reduced lesional expression of TSLP, leading to reduced aggregation of macrophages and M2 macrophages, and of Tregs, Th2, and Th17 cells but an increased aggregation of Th1 cells, in lesions, which, in turn, yields retarded fibrogenesis. Similarly, Tregs depletion resulted in suppression of platelet aggregation, and reduced aggregation of M2 macrophages, Th2, and Th17 cells but increased aggregation of Th1 cells, in lesions. In addition, both platelet and Tregs depletion resulted in depressed TGF-β1/Smad3 and PDGFR-β/PI3K/Akt signaling, leading to arrested EMT, FMT, and fibrogenesis. Taken together, our study highlights the critical roles of immune cells in general and platelets and Tregs in particular in lesional development and fibrogenesis, and suggests that the type 2 immunity may play a vital role in lesional fibrogenesis. In addition, our study provides more credence to the ReTIAR notion, since, through the ReTIAR lens, we can coherently piece together seemingly unrelated findings, such as TSLP overexpression, M2 macrophages, platelet aggregation, and the Th1/Th2 imbalance in endometriosis.

Our results are consistent with the burgeoning and overwhelming data in support for a predominantly type 2 immunity in lesional microenvironment (18, 20, 21, 54). Indeed, levels of Th2 cytokines, such as IL-4 (55–57), IL-13 (58), IL-25 (59), and IL-33 (60–63), in lesions or the peritoneal fluid from women with endometriosis have been reported to be elevated. IL-4 can stimulate the proliferation of endometriotic stromal cells (57), and, in conjunction with PGE_2_, may enhance estrogen production in endometriotic tissues (64). IL-33 treated mice with induced endometriosis are highly vascularized and exhibited increased proliferation (62). In contrast, IL-12 has been shown to inhibit lesional development (36, 65), so have IFN-γ and IL-2 (21). In addition, macrophages infiltrated in lesions are found to be polarized to alternatively activated or M2 macrophages (39, 66, 67). In particular, M2a macrophages, which can be induced by IL-4 or IL-13—two typical Th2 cytokines—have been reported to be critically involved in fibrogenesis of endometriosis (8). Our findings are in broad agreement with a preponderance of published data indicating more Tregs infiltration in endometriotic lesions (68, 69), especially in deep endometriotic lesions (70). They also are in line with the reported higher concentrations of IL-17 in peritoneal fluid or plasma from women with endometriosis (71–73), which is found to trigger proinflammatory cytokines and angiogenetic growth factors (73). In particular, they are consistent with the increase in Tregs, Th2, and Th17 cells in endometriosis (20, 74–76). Tregs secreted fibrinogen-like protein 2 (FGL-2) can promote the production of Th2 cytokines, with concomitant inhibition of Th1- and Th17-oriented immunity (77). Tregs could also induce human monocytes to differentiate into M2-like macrophages (74).

Our study is also consistent with our previous report that endometriotic lesions exhibited progressive cellular changes consistent with the progressive EMT, FMT, SMM, and fibrogenesis, while antiplatelet treatment resulted in significant hindrance to EMT, FMT, SMM, and fibrogenesis and reduced lesion weight (34). Platelet granules contain a variety of cellular growth factors and cytokines (78, 79), such as TGF-β1, PDGF, and vascular endothelial growth factor (VEGF), that may accelerate lesional development and fibrogenesis (80). In particular, activated platelets can release TGF-β1 and induce the TGF-β/Smad signaling pathway, promoting EMT, FMT, and SMM in lesions, resulting ultimately in fibrosis (3).

TSLP is known to promote the differentiation of naïve T-cells into a Th2 phenotype, a type 2 immune response, and the secretion of various profibrotic factors in DCs dependent or independent manner (16, 26, 32, 33, 81–83). Incidentally or not, DCs have been reported to be significantly increased in peritoneal endometriotic lesions (84), which may promote angiogenesis and lesion growth in endometriosis (85). Plasmacytoid DCs are reported to facilitate endometriosis development through angiogenesis during the early disease stage by secreting IL-10 (86). TSLP induces cytokine production by human ILC2s, reinforces GATA3 expression, and therefore helps to sustain IL-33-induced activation (87). IL-33 stimulated ILC2s to interact through inducible T cell costimulator-ligand (ICOS-L) with ICOS expressed by Tregs, and this interaction is needed for IL-33-mediated induction of Tregs (88). TSLP may also promote M1 to M2 macrophage polarization (89).

Platelets can secrete a copious amount of TGF-β1, which plays an important role in promoting the differentiation of Tregs and Th17 (90). TSLP has been reported to drive the proliferation of cutaneous Tregs (91). Mucosal DCs induced by various cytokines, including TSLP, have been reported to induce polarization of T cells toward a Th2 response, and the differentiation of Tregs (92). TSLP is also reported to act directly on CD4 single-positive thymocytes to promote Tregs differentiation (93). Our study found that with the progression of endometriosis, the density of Tregs in the lesions increased gradually and progressively, and their density was positively correlated with the extent of fibrosis in lesions, demonstrating that Tregs promote, at least in part, the progression and fibrogenesis of endometriosis. After platelet depletion, the density of Tregs in endometriotic lesions remained at a low level 1, 2, and 5 weeks after the induction of endometriosis, which was significantly lower than that in Control mice. This suggests that platelets can promote the proliferation or recruitment of Tregs in endometriosis, possibly through the secretion of TGF-β1 and the up-regulation of TSLP expression in endometriotic lesions. Endometriotic stromal cells themselves can also secrete platelet-activating molecules (94). In addition, our study showed that Tregs depletion resulted in smaller lesions, which may consequently either reduce the amount of platelet-activating molecules secreted by endometriotic cells or lead to a reduced number of potentially activatable platelets due to smaller lesions. In either case, this would yield the net effect of decreased platelet activation.

Endometriosis is often said to have enigmatic pathogenesis and pathophysiology. It has been traditionally viewed as an estrogen-dependent disease, as well as an inflammatory condition (95). So why the type 1 immunity, which is characterized by inflammation, can be a hindrance to lesional progression and fibrogenesis while the type 2 immunity can be a promoter?

First of all, after each cyclic bleeding, endometriotic epithelial and stromal cells may secrete alarmins such as IL-25, IL-33, and TSLP (96). In fact, endometriotic stromal cells themselves can secrete platelet-activating molecules (94). Activated platelets can induce estrogen production in endometriotic stromal cells (97, 98), which, in turn, may induce TSLP secretion (30). Through activation of DCs, TSLP can promote Th2 cell differentiation and propagate a type 2 immune response (16). Similarly, IL-33, which is in high concentration in endometriosis (60, 61, 63, 99), can also induce a Th2 response on mast cells and Th2 cells (16). Th2 cell-derived IL-4 and IL-13 can alternatively activate macrophages (100) and suppress Th1 cells, resulting in increased Th2 cytokine production. Th2 cells, along with M2 macrophages, eosinophils, basophils, and ILC2s, promote the type 2 immunity-induced fibrogenesis (16). This seems to occur exactly in endometriosis (62, 99).

Second, IFN-γ secreted by Th1 cells mediates the activation of classical or M1 macrophages (101–103), which express Th1 and Th17-polarizing cytokines IL-12, IL-23, IL-27, and Th1-recruiting chemokines CXCL9, CXCL10, and CXCL11. In contrast, M2 macrophages support the resolution of inflammation by the expression of anti-inflammatory molecules such as IL-10, TGF-β, and IL-1Ra (104). M2 macrophages can also recruit Th2 and Tregs through secretion of the CCL17 and CCL1 chemokines (105). Th1 cells and M1 macrophages are mutually promotional, so are Th2 cells and M2 macrophages (104). Hence, the presence of Th2 cytokines would further promote the lesional dominance of a type 2 immunity.

Both IL-12 and IFNγ can induce the differentiation of naïve CD4 cells to Th1 cells to produce the pro-inflammatory cytokine IFNγ (106), which reduces collagen production and fibronectin expression (107, 108). In contrast, Th2 cytokine IL-4 increases collagen production (108). This may explain as why IL-12 inhibits lesional development (36, 65), so do IFN-γ and IL-2 (21).

Third, Th17 cells that express the proinflammatory cytokine IL-17A is an important driver of fibrosis. IL-17A expression has been implicated in the pathogenesis of pulmonary fibrosis, chronic allograft rejection, fibrosis in orthotopic lung transplantation, myocardial fibrosis, and hepatitis-induced hepatic fibrosis (109, 110).

Fourth, Tregs also participate in tissue repair, mainly through macrophage polarization, since they secrete IL-10, IL-4, and IL-13, which induces the transition to M2 macrophages (111). M2 macrophages reduce the inflammatory cytokine secretion, and their activity is converted into regenerative activity, which is critical for tissue repair (101, 112, 113).

**Figure 8:**
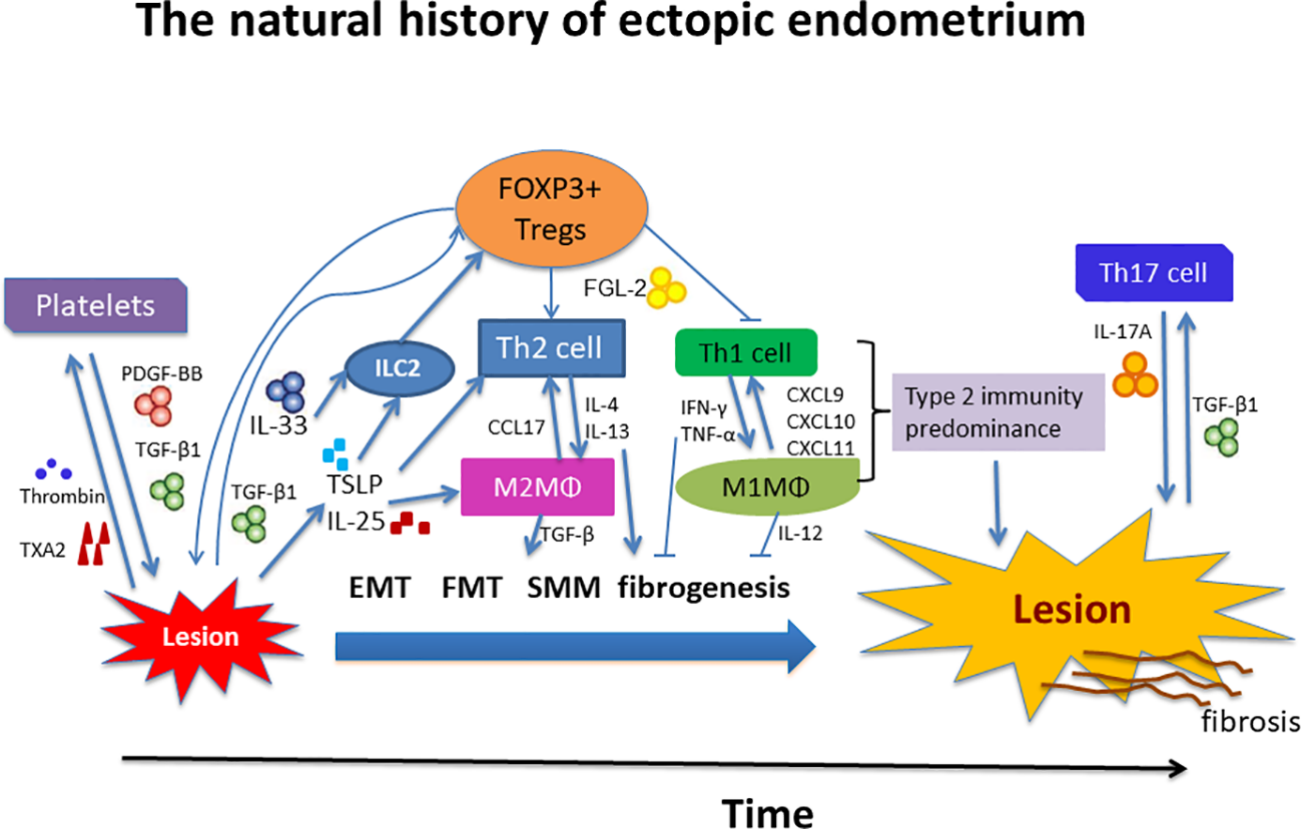
Schematic summary of local immune environment during progression of endometriosis. Tissue injury results in the release of alarmins such as IL-25, IL-33, and TSLP, which, individually or collectively, promote a type 2 immune response. In particular, platelets and Tregs together promote the Type 2 immunity predominance, which is exemplified by recruitment and aggregation of of Th2 cells, M2 macrophages, and possibly group 2 innate lymphocytes (ILC2s) in the lesional immune microenvironment. These type 2 immune cells subsequently release type 2 cytokines, such as IL-4 and IL-13, which polarizes macrophages into alternatively activated M2 macrophages. M2 macrophages can release copious TGF-β1 and PDGF, inducing EMT, FMT, SMM, and fibrogenesis. Platelets and Tregs, in and by themselves and also by the induction of a type 2 immune response, induce the TGF-β1 and PDGF signaling pathways to promote fibrogenesis in endometriosis. See text for more details. IL, interleukin; PDGF-BB, platelet-derived growth factor-BB; ILC2, group 2 innate lymphoid cells; MΦ, macrophage; Tregs, regulatory T cells; Th, T helper cell; TGF-β1, transforming growth factor β1; IFN-γ, interferon-γ; TNF-α, tumor necrosis factor-α; CCL17, CC chemokine ligand 17; CXCL, CXC chemokine ligand; FGL-2, fibrinogen-like protein 2; TXA_2_, thromboxane A2; TSLP, thymic stromal lymphopoietin; EMT, epithelial-mesenchymal transition; FMT, fibroblast-to-myofibroblast transdifferentiation; SMM, smooth muscle metaplasia.

We summarize this line of reasoning in **Figure 8**. We note that many seemingly isolated findings can be organically and coherently integrated into this ReTIAR framework, which is essentially a sketch of a roadmap for future studies. This is important, since without a roadmap, we can only see the tree leaves or branches, or at most isolated trees, but not the forest. We can only grope, akin to blind men trying to figure out what an elephant looks like. Indeed, with this roadmap, perhaps we can also investigate other suspected players, such as ILC2s, IL-25, TSLP, DCs, eosinophils, and basophils in the future.

This roadmap also has important clinical implications as well. For example, we can now understand why the trial on anti-TNF-α therapy failed (114), since TNF-α is a Th1 cytokine. We can also understand why a therapy based on the recombinant IL-2, a Th1 cytokine, which was launched ostensibly based on its putative anti-inflammatory propensity, failed in endometriosis (115). This is because Tregs constitutively express high levels of CD25, an IL-2 receptor, and are easily expanded using continuous low levels of IL-2. In addition, pentoxifylline also failed in clinical trials (116), since pentoxifylline suppresses the production of TNF-α and IFN-γ while increases the production of Th2 cytokines (117). Clearly, the elucidation of the lesional immune microenvironment should guide us to choose the best therapeutics while avoid the ineffective ones.

This study has several strengths. First, by employing a serial design and by depleting platelets and/or Tregs, we have unveiled the facilitative roles of both platelets and Tregs in lesional progression and fibrogenesis. In particular, we have demonstrated the time-dependent nature of several cell types (Th1 *vs.* Th2 cells, for example). This time dependency may explain as why we often see conflicting reports in the literature, aside from the difference in methodology and sample sizes. Second, by embedding our experiments within the ReTIAR framework or as a backbone, which allows us to stand on the shoulders of giants who have advanced our knowledge on wound healing, we were able to see a much bigger picture on how lesions progress. This should help us and our fellow investigators to see the forest, instead of each individual leaves or trees, so that many seemingly isolated findings can be pieced together. Third, by depleting platelets and Tregs separately or jointly, our data suggested that platelets played a more active role in inducing TSLP expression, which is crucial in inducing a type 2 immunity and in facilitating the proliferation of Tregs. Lastly, we allowed a sufficiently long observation period after endometriosis induction to investigate the role of platelets and Tregs in fibrogenesis. Collectively, these complementary approaches provide more credible data.

This study also has several limitations. First, our study is limited by the use of histologic and immunohistochemistry analyses only. It lacks confirmation by flow cytometry on changes in the density of related immune cells in circulation after platelet or Tregs depletion, which may show the systemic immunological alterations rather than just within endometriotic lesions alone. In addition, there is a lack of molecular data that can provide a more definitive proof for the involvement of signaling pathways, or to elucidate the molecular mechanisms on how platelets and Tregs interact with each other. This should await for more research in the future. Second, to conform with the platelet depletion groups, we used the anti-CD25 antibody for Tregs depletion, instead of using the transgenic mouse expressing a diphtheria toxin receptor-GFP fusion protein under the control of the FOXP3 locus for the Tregs depletion, which should be more specific in terms of Tregs depletion (118). CD4+ Tregs can be identified by their constitutive expression of CD25. However, the existence of CD4+ CD25^−^ Tregs and the expression of CD25 in activated conventional T cells preclude the discrimination between Tregs and activated conventional T cells, which may limit the interpretation of data obtained by the use of anti-CD25-depleting antibodies (119–123). Third, we depleted platelets or Tregs but did not transfer platelets or Tregs back into the mice. Hence, in essence, we have “loss of function” data but no “gain of function” data. Of course, platelet infusion appears to accelerate fibrogenesis of endometriosis in mouse (5). Further mechanistic research is warranted in the future.

In summary, our study demonstrates that both platelet and Tregs depletion decelerated lesional progression and fibrogenesis by disrupting predominantly a type 2 immune response in lesional microenvironment. It suggests that in the course of lesional progression, platelets may induce TSLP, leading to a type 2 immune response in the lesional microenvironment, and, ultimately, to fibrosis if unimpeded. Taken together, our study highlights the critical roles of immune cells in general and platelets and Tregs in particular in lesional development and fibrogenesis, and suggests the type 2 immunity may play a vital role in lesional fibrogenesis. In addition, our study provides more credence to the ReTIAR notion, since, thorough the ReTIAR lens, we can coherently piece together seemingly isolated findings, such as TSLP overexpression, M2 macrophages, platelet aggregation, and the Th1/Th2 balance.

## Data Availability Statement

The raw data supporting the conclusions of this article will be made available by the authors, without undue reservation.

## Ethics Statement

The animal study was reviewed and approved by the Institutional Experimental Animals Review Board of Shanghai OB/GYN Hospital, Fudan University.

## Author Contributions

S-WG conceived the study, analyzed the data, and drafted the manuscript. FX conducted the experiments, analyzed the data, and wrote the manuscript. S-WG and XL contributed reagents, conceived the experiments, provided financial support, and edited the manuscript. All authors contributed to the article and approved the submitted version.

## Funding

This research was supported in part by grants from the National Science Foundation of China (81530040 and 81771553 to S-WG; 81671436 and 81871144 to XL), and an Excellence in Centers of Clinical Medicine grant (2017ZZ01016) from the Science and Technology Commission of Shanghai Municipality.

## Conflict of Interest

The authors declare that the research was conducted in the absence of any commercial or financial relationships that could be construed as a potential conflict of interest.
